# Descriptive Analysis of Inpatient Pharmacist Interventions at a Tertiary Care Military Hospital in Eastern Province, Saudi Arabia

**DOI:** 10.7759/cureus.77926

**Published:** 2025-01-24

**Authors:** Saleh H Almutairi, Wafa K Alanazi, Rahayef N Alotaibi, Manar H Alonayzan

**Affiliations:** 1 Department of Hospital Material and Management, King Fahd Military Medical Complex, Dhahran, SAU; 2 Department of Pharmaceutical Services, King Fahd Military Medical Complex, Dhahran, SAU; 3 Department of Pharmacy Practice, King Faisal University, Al Ahsa, SAU

**Keywords:** inpatient pharmacy, inpatient units, medications errors, pharmacist interventions, saudi arabia

## Abstract

Background

Medication errors can be categorized into five main categories: prescribing, compounding, dispensing, distribution, and administration. Prescribing errors, including wrong indication, dose, frequency, and route of administration, are the most prevalent preventable medication errors. Over the past decades, pharmacists' role in minimizing such errors has grown with the development of pharmaceutical care. Thus, emphasizing pharmacists' interventions regarding prescribed medications is essential.

Aims

This study evaluated the frequency and types of medication errors addressed by in-patient pharmacists, as well as the acceptance of these interventions by physicians at King Fahd Military Medical Complex (KFMMC) in Dhahran, Saudi Arabia.

Methods

This is a retrospective study that analyzed data on inpatient pharmacists' interventions at KFMMC. All inpatient prescriptions from January to December 2022 were involved in the study. Primary data collected from the inpatient pharmacy system include pharmacist intervention type, doctor's action, ward, dosage, medication classification, and whether it falls under high-alert medication. Data was tabulated in Excel (Microsoft, Redmond, WA, USA) and analyzed using SPSS version 27 (IBM Corp., Armonk, NY, USA).

Results

A total of 9594 pharmacy interventions were analyzed. Approximately 1300 (13.6%) of the pharmacist interventions were approved by the physicians. The rate of clinical intervention was 563 (5.9%). More than half of the interventions were from the specialty ward (N=5261; 55.1%). The intervention rate for intravenous dosage was 4837 (50.4%), and 2028 (21.1%) of the medication was considered high alert. The top three most common medication interventions were related to nutrition and blood (N=2268; 23.6%), followed by antimicrobial medications (N=2243; 23.4%) and gastrointestinal medications (N=1731; 18%). Moreover, most of the clinical interventions were related to antimicrobial medicines. No significant relationships were observed between the medication classes and the doctor’s action (p=0.087).

Conclusion

This study highlights the critical role of pharmacist intervention in reducing medication errors. Nutrition and blood medications were most prescribed due to the critical conditions of hospitalized patients, while antimicrobial prescriptions required pharmacist input to optimize therapy and address drug resistance. Pharmacist intervention detected significant errors such as wrong dose, duplication, and frequency, improving patient outcomes and collaboration with physicians, ultimately enhancing healthcare quality at KFMMC. Future research should analyze outpatient pharmacist interventions and strategies for addressing pending interventions.

## Introduction

Medication errors are any avoidable event that can result in or lead to improper medication use or patient harm while the medication is controlled by the healthcare professional, patient, or consumer [1].

Medication errors can be categorized into five main categories: prescribing, compounding, dispensing, distribution, and administration [2]. Prescribing errors are the most common type of medication error, including wrong indication, dose, frequency, or route of administration [2]. Alongside this, it is also the common cause of morbidity and mortality in community practice and hospitals, leading to an economic burden on the healthcare system in Saudi Arabia [3]. The World Health Organization (WHO) reports that medication errors harm 1.3 million individuals every year and result in one fatality daily in the United States [4]. WHO also approximates the worldwide cost of medication errors to be around $42 billion annually [4].

Fortunately, such medication errors are preventable, and the significant presence of pharmacists can substantially relieve healthcare costs [5]. Over recent decades, the pharmacists’ role has evolved with the development of pharmaceutical care. Pharmacists can actively collaborate in patient care with doctors and other healthcare professionals [6]. The involvement of pharmacists in patient care is beneficial in reducing adverse drug events, shortening hospital stays, and lowering mortality rates [7]. For these benefits to be achieved, a judicious medicine use process, from choosing the right medicine and dose to supporting patient counseling by the health care providers, would play a significant role [7].

Several studies highlighted the importance of pharmacists' interventions in hospital settings, emphasizing that prescribing errors occurred commonly and that pharmacists' interventions were critical in preventing possible medication-related harm to patients [8]. In addition, another study described the importance of the pharmacists' role in detecting, reporting, and reducing prescription-related errors in different hospital wards, such as intensive care units, neonatal intensive care units, pediatric intensive care units, critical care units, and heart failure clinics [2,9,10].

However, studies are still needed to analyze and assess pharmacist interventions in Saudi Arabia, considering factors such as hospital wards and drug classes, which can provide information about the most and least hospital wards benefiting from pharmacist interventions and the most drug classes associated with prescribing errors. Due to the lack of research on pharmacist interventions in military hospitals in Saudi Arabia, this work will highlight the role of inpatient pharmacists in reducing medication errors through interventions among several wards at King Fahd Military Medical Complex (KFMMC).

## Materials and methods

This is a retrospective study conducted from January to December 2022 at the pharmacy department of KFMMC-Dhahran, Saudi Arabia. The study was approved before data collection by the Institutional Review Board of Armed Forces Hospitals Eastern Province, Saudi Arabia (no. AFHER-IRB-2024-021). Our analysis focuses only on inpatient pharmacist interventions. Hence, the study included all data from inpatient prescriptions with pharmacist interventions during 2022, excluding outpatient prescriptions. Data collected from the inpatient pharmacy system include pharmacist intervention type (clinical and non-clinical). Clinical interventions include dose, duplication, and frequency, whereas non-clinical interventions include Instruction, availability, and wrong order (order from intravenous and unit dose). In the hospital’s e-prescribing system, which was developed internally by the hospital's engineers, the ordering of medications is organized into two distinct pathways. Physicians can select the ‘intravenous’ section to order intravenous medications, while they use the ‘unit dose’ section to order oral medications. A wrong order entry occurs when a physician mistakenly orders intravenous medications in the unit dose section and vice versa. The collected data also contains doctors' actions (approved, disapproved, and pending), hospital wards (specialty, surgical, pediatric, medical, intensive care units, obstetrics/gynecology, and accidental and emergency), dosage form (intravenous and unit dose), medication class (nutrition and blood, antimicrobial, medications, gastrointestinal medications, cardiovascular medications, nervous system medications, endocrine medications, respiratory medications, skin medications, musculoskeletal and joint medications, eye medications, immunological products and vaccines, obstetrics, gynecology, and urinary tract disorder, malignant disease and immunosuppression, miscellaneous, ear, nose and oropharynx, anesthesia medications and antidote) and whether the medication considers a high alert or not, such as concentrated electrolytes, antithrombotic agents, anticholinergic drugs, anesthetic agents, local anesthesia, and hazardous/cytotoxic drug.

Descriptive statistics were described as numbers and percentages for all categorical variables. The association between the type of intervention and doctoral action among the medical class was conducted using the Chi-square test. Statistical significance was set to p<0.05 level. All statistical data were analyzed using SPSS version 27 (IBM Corp., Armonk, NY, USA).

## Results

A total of 9594 inpatient pharmacist interventions during 2022 were analyzed. As seen in Table 1, 563 (5.9%) of the pharmacist interventions had clinical intervention. Among the clinical interventions, the most common was medication frequency (N=262; 2.7%). For non-clinical intervention, 81 (0.9%) were wrong order entries (order from intravenous and unit dose). Approximately 1300 (13.6%) of the pharmacist interventions were approved by the physicians. The most common ward that occurred pharmacist intervention was the specialty ward (male specialty) (N=5287; 55.1%), followed by the medical ward (cardiac ward, female or male ward) (N=1142; 11.9%), and surgical ward (female or male surgical, day case) (N=1091; 11.4%). Approximately half (N=4837; 50.4%) were related to intravenous dosage, and 2028 (21.1%) were considered high-alert medication.

**Table 1:** Descriptive analysis of inpatient pharmacy intervention (n=9594).

Study variables	N (%)
Type of Intervention	
· Clinical	563 (05.9%)
· Non-clinical	9031 (94.1%)
Pharmacist clinical intervention	
· Dose	90 (0.90%)
· Duplication	209 (02.2%)
· Frequency	262 (02.7%)
Pharmacist non-clinical intervention	
· Other not classified	6312 (65.8%)
· Wrong order entry	81 (0.90%)
· Not available	2640 (27.5%)
Doctors' actions	
· Approved	1300 (13.6%)
· Disapproved	133 (01.4%)
· Pending	8161 (85.1%)
Hospital wards	
· Specialty	5287 (55.1%)
· Surgical	1091 (11.4%)
· Pediatric	503 (05.2%)
· Medical	1142 (11.9%)
· Intensive care units	620 (06.5%)
· Obstetrics/gynecology	302 (03.1%)
· Accidental and emergency	649 (06.8%)
Dosage form	
· Intravenous	4837 (50.4%)
· Unit dose	4757 (49.6%)
Medication classified as high alert	
· Yes	2028 (21.1%)
· No	7566 (78.9%)

**Figure 1:**
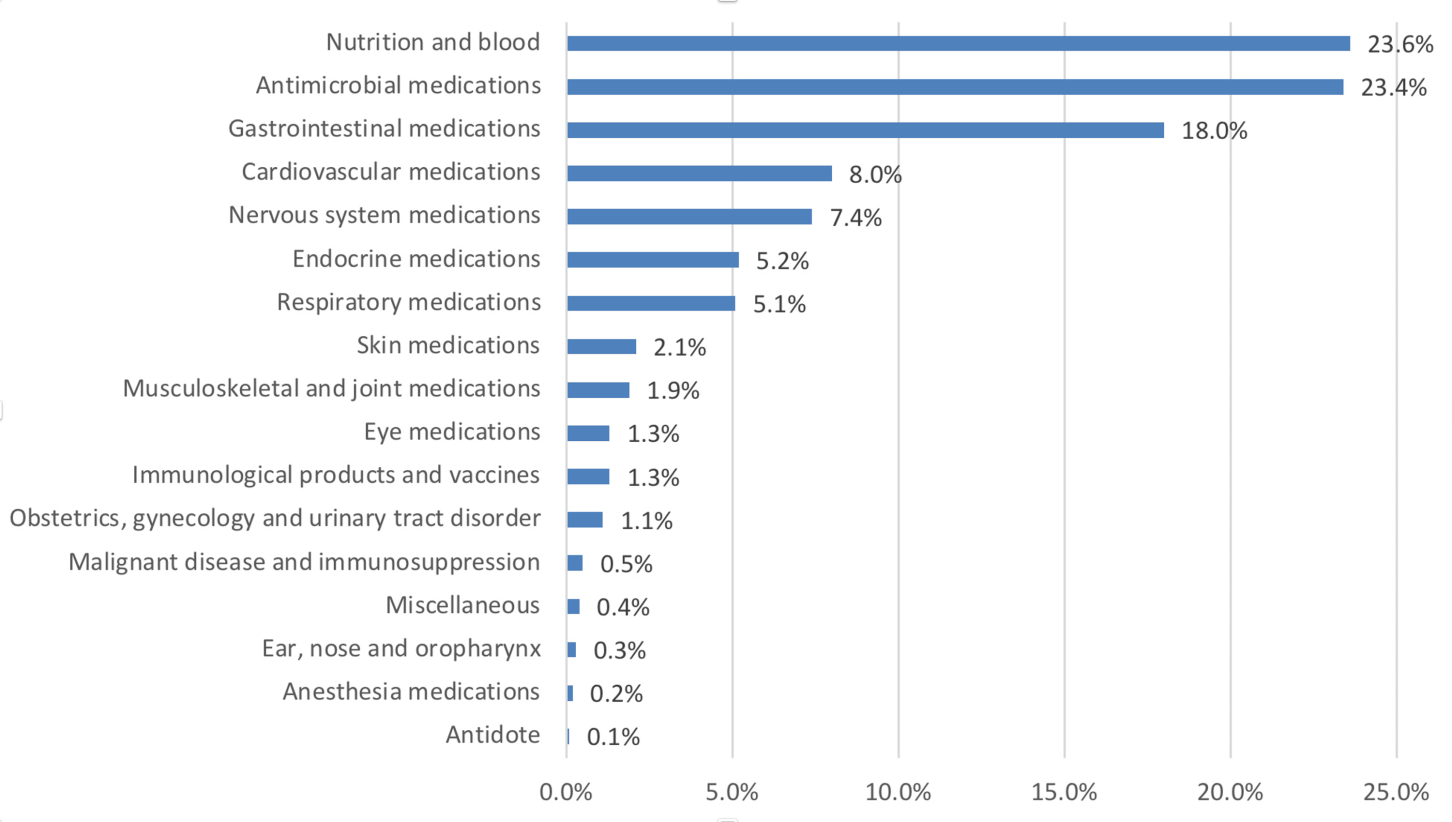
Distribution of medication classes in general

In addition, Figure 1 shows the top five most commonly prescribed medications that had pharmacist interventions: nutrition and blood medications (N=2268; 23.6%), such as fluids/electrolytes, intravenous nutrition, minerals and vitamins, followed by antimicrobial medications (N=2243; 23.4%), gastrointestinal medications (N=1731; 18%), cardiovascular medications (N=765; 8%), and nervous system medications (N=709; 7.4%), while antidote recorded the least (N=10; 0.1%).

Moreover, the relationship between the intervention type and medication class is observed In Table 2. Antimicrobial medications 249 (44.2%) were primarily associated with the clinical type of intervention (p<0.001), while nutrition and blood 2210 (24.5%), antimicrobial medications 1994 (22.1%), and gastrointestinal medications 1639 (18.1%) were associated with non-clinical interventions (p<0.001).

**Table 2:** The relationship between medication classes and the type of pharmacist intervention (n=9594). _§ P-value has been calculated using the Chi-square test._ _ ** Significant at p<0.05 level._

Medication	Type of intervention		P-value §
	Clinical N (%) (n=563)	Non-Clinical N (%) (n=9031)	
Antimicrobial medications	249 (44.2%)	1994 (22.1%)	<0.001 **
Cardiovascular medications	57 (10.1%)	708 (07.8%)	
Endocrine medications	13 (02.3%)	490 (05.4%)	
Gastrointestinal medications	92 (16.3%)	1639 (18.1%)	
Immunological products and vaccines	04 (0.70%)	121 (01.3%)	
Eye medications	02 (0.40%)	121 (01.3%)	
Malignant disease and immunosuppression	02 (0.40%)	48 (0.50%)	
Miscellaneous	0	36 (0.40%)	
Musculoskeletal and joint medications	25 (04.4%)	158 (01.7%)	
Nervous system medications	47 (08.3%)	662 (07.3%)	
Nutrition and blood	58 (10.3%)	2210 (24.5%)	
Obstetrics, gynecology and urinary tract disorder	05 (0.90%)	104 (01.2%)	
Respiratory medications	07 (01.2%)	486 (05.4%)	
Skin medications	02 (0.40%)	199 (02.2%)	
Ear, nose and oropharynx	0	29 (0.30%)	
Anesthesia medications	0	16 (0.20%)	
Antidote	0	10 (0.10%)	

Last, Table 3 reveals no statistical variation between the medication classes and doctors' actions (p=0.087).

**Table 3:** This table reveals no statistical variation between the medication classes and doctors' actions (p=0.087). _§ P-value has been calculated using the Chi-square test._

Medication	Doctor action		P-value §
	Approved N (%) (n=1300)	Disapproved N (%) (n=133)	
Antimicrobial medications	445 (34.2%)	52 (39.1%)	0.087
Cardiovascular medications	65 (05.0%)	04 (03.0%)	
Endocrine medications	15 (01.2%)	04 (03.0%)	
Gastrointestinal medications	193 (14.8%)	11 (08.3%)	
Immunological products and vaccines	06 (0.50%)	01 (0.80%)	
Eye medications	03 (0.20%)	0	
Malignant disease and immunosuppression	01 (01.0%)	01 (0.80%)	
Miscellaneous	03 (0.20%)	0	
Musculoskeletal and joint medications	26 (02.0%)	0	
Nervous system medications	20 (01.5%)	01 (0.80%)	
Nutrition and blood	517 (39.8%)	59 (44.4%)	
Obstetrics, gynecology and urinary tract disorder	06 (0.50%)	0	

## Discussion

Pharmacists' intervention is essential in clinical outcomes, particularly when integrated into patient care teams. The education provided by the pharmacist among patients could result in better adherence to medication, streamlining complex regimens, and addressing side effects. Hence, this study will provide more insights into the impact of pharmacist medication interventions in a tertiary healthcare setting. This study finds that pharmacist interventions resulted in the clinical detection of medication frequency (N=262; 2.7%), duplication (N=209; 2.2%), and improper dosage (N=90; 0.9%). Consistent with our findings, several studies documented that pharmacist intervention yielded significant uncovering of PEs, including incomplete orders, frequency, wrong doses, unauthorized prescriptions, and medication discontinuation [3,6,9-12]. This study emphasized that the pharmacist's role in a multidisciplinary team is vital. Pharmacists may help reduce adverse events, enhance patient safety and understanding, prevent readmission, and decrease the duration of hospitalization [7]. In contrast, the lack of pharmacist interventions could lead to more PEs due to a lack of adherence to prescribing guidelines or other organizational aspects, including inadequate training, lack of interdisciplinary medical teams, and lack of attention to self-awareness of errors [13].

The collaboration between physicians and pharmacists can help optimize patient outcomes. Our study finds that the physicians approved 13.6% (N=1300) of pharmacist interventions, and only 1.4% (N=133) disapproved. Most interventions were pending for the doctor's action (85.1%), possibly due to time constraints, urgency, resource limitations, and individual doctor preferences. In Taif, Saudi Arabia, physicians accepted 370 out of 404 pharmacist interventions (91.6%), a rate higher than reported in our study [10]. Supporting this finding, the acceptance rate of pharmacist interventions at Jazan General Hospital was 82.5% in 2016 and 2017 [8]. This aligns with findings from a study conducted in the USA [7]. The differences in results could be due to the study methodology, number of cases, and year of study. However, other factors, including regional settings and study duration, might also contribute to this effect.

Most of the PEs were related to the specialty ward (N=5257; 55.1%), followed by the medical ward (N=1142; 11.9%) and surgical ward (N=1091; 11.4%). Of all the interventions (N=9594), 21.1% (N=2028) were considered medication high alert, such as concentrated electrolytes, antithrombotic agents, anticholinergic drugs, anesthetic agents, local anesthesia, and hazardous/cytotoxic drugs. A retrospective study identified the intensive care unit as the leading site for prescribing errors, followed by the neonatal intensive care unit, critical care unit, and pediatric intensive care unit [3]. These results align with a prospective study conducted in Jazan, Saudi Arabia, which found that the highest rate of drug-related problems (DRPs) was reported in the pediatric intensive care unit, followed by the neonatal intensive care unit and pediatric emergency department [9]. These variation rates of DRPs could be linked to pathologies, duration of hospital stay, and the lack of pharmacists in the pediatric intensive care unit compared to other hospital units [9].

The majority of the PEs were associated with nutrition and blood medications (N=2268; 23.6%), possibly due to the critical nature of hospitalized patients, which requires careful management of conditions like anemia, coagulopathies, or malnutrition, particularly with high-risk drugs such as anticoagulants. Antimicrobial medications come second (N=2243; 23.4%) because this class requires pharmacist interventions to optimize therapy, address drug resistance, and adjust doses in complex cases. They are followed by gastrointestinal medications (N=1731; 18%), cardiovascular medications (N=765; 8%), and nervous system medications (N=709; 7.4%). Data further suggest that clinical interventions were prevalent in antimicrobial medications (p<0.001). However, the doctor's actions in these medication interventions yielded no significant differences (p=0.087). A limited sample size in some key categories may have influenced this result. Hence, further investigations are warranted to determine its effect. This is broadly consistent with the findings of a retrospective review study conducted in a general tertiary care hospital in Qatar [12]. The pharmacological classes associated with interventions were anti-infective and cardiovascular medications. Supporting these findings, it was documented that anti-infective, alimentary tract, and metabolism medications were the classes most commonly associated with prescribing errors [11]. This contradicted previous reports, which indicated that, between 2016 and 2017, antibiotics were the most frequently reported drug-related problems, followed by proton pump inhibitors and statins [8]. These differences were likely due to study settings, population studies, data collection methods, periods, and healthcare provider knowledge. 

This study has several limitations. First, it is a retrospective descriptive design that focuses on inpatient pharmacist interventions only, which suggests that multi-center studies should be conducted to understand better the generalizability of pharmacist interventions across various hospital settings. Additionally, we could not assess doctors' responses to specific pending interventions, which may underestimate the efficacy of pharmacist interventions. In addition, some key comparisons have limited cases, particularly regarding the type of intervention and doctor action about medication class. Hence, we cannot generalize the pairwise comparison of these variables and may not further investigations.

Future studies should evaluate strategies for addressing pending and assessing pharmacist interventions in outpatient settings. They should also consider their impact on vulnerable populations, such as pediatric, geriatric, or critically ill patients, where medication errors could have more severe consequences. Finally, they should assess the cost-effectiveness of pharmacist interventions in reducing healthcare costs associated with adverse drug events, hospital readmissions, and extended lengths of stay.

## Conclusions

This study provides evidence that pharmacist intervention plays a significant role in reducing medication errors in healthcare settings. In this study, nutrition and blood medications were the most commonly prescribed due to the critical nature of hospitalized patients, requiring careful management of their conditions. Prescription of antimicrobial medications was prevalent during clinical interventions because such medications require pharmacist interventions for optimizing therapy, addressing drug resistance, and adjusting doses in complex cases. Generally, pharmacist intervention could detect some clinically significant PEs, including wrong dose, duplication, and frequency. Detection of these PEs could enhance patient outcomes and improve collaborative care between physicians and pharmacists. This ultimately leads to higher healthcare quality at KFMMC. Further studies should focus on analyzing pharmacist interventions in outpatient settings and evaluating strategies for addressing pending interventions.
